# Q fever: A neglected disease of camels in Giza and Cairo Provinces, Egypt

**DOI:** 10.14202/vetworld.2019.1945-1950

**Published:** 2019-12-12

**Authors:** Hend H. A. M. Abdullah, Hany A. Hussein, Khaled A. Abd El-Razik, Ashraf M. A. Barakat, Yousef A. Soliman

**Affiliations:** 1 Department of Parasitology and Animal Diseases, National Research Centre, Dokki, Giza, Egypt; 2 Department of Animal Reproduction and Artificial Insemination, National Research Centre, Dokki, Giza, Egypt; 3 Key Laboratory of Diagnostic and Detective Technology, Department of Veterinary Research, Guangdong Haid Institute of Animal Husbandry and Veterinary, Guangzhou, China; 4 Department of Zoonotic Diseases, National Research Centre, Dokki, Giza, Egypt; 5 Department of Biotechnology, Central Laboratory for Evaluation of Veterinary Biologics, Abbasia, Cairo, Egypt

**Keywords:** camel, *Coxiella burnetii*, enzyme-linked immunosorbent assay, standard polymerase chain reaction, trans-quantitative polymerase chain reaction

## Abstract

**Background and Aim:**

Q fever is a zoonotic disease caused by *Coxiella burnetii*. Cattle, sheep, and goat are the main reservoir of *C. burnetii*. In Egypt, the epidemiological data about *C. burnetii* in camels are limited. Therefore, the current study was conducted to identify *C. burnetii* infection in camels by different molecular tools and to estimate its seropositivity through the detection of anti-C. burnetii antibodies in camel sera.

**Materials and Methods:**

Blood samples were collected 112 from camels in Giza and Cairo Provinces, Egypt. All blood samples were screened by trans-quantitative polymerase chain reaction (trans-qPCR) for *C. burnetii* and positive samples subjected to standard PCR using the superoxide dismutase enzyme coding gene of *C. burnetii*. Sera of studied camels were examined for the presence of antibodies against *C. burnetii* using enzyme-linked immunosorbent assay.

**Results:**

Out of 112 camels, 19 were positive for *C. burnetii* by qPCR with an overall prevalence of 16.9% (18.6% in Giza and 15.1% in Cairo Provinces, respectively). The seroprevalence of anti-*C. burnetii* IgG antibodies in the examined camels was 4.5% (5/112).

**Conclusions:**

Trans-qPCR assay is a rapid and sensitive tool for the detection of *C. burnetii* in acute stage. Camels should be considered one of the major reservoirs for *C. burnetii* in Egypt.

## Introduction

Q fever is an acute, highly contagious zoonotic disease that is commonly neglected [1]. It is caused by *Coxiella burnetii*, a strict intracellular Gram-negative bacterium [2]. *C. burnetii* has been classified by Centers for Disease Control and Prevention as a potential bioterrorism agent [3]. The organism can infect a wide variety of animals, human, birds, and arthropods; however, ruminants act as the main reservoir [1]. Q fever infection in animals is mostly clinically inapparent; nonetheless, abortion, stillbirth, decrease in the reproduction efficiency, and infertility are all reported [4]. In human, the acute *C. burnetii* infection is characterized by fever, flu-like signs, headache, and pneumonia, whereas hepatitis and endocarditis are serious complications in chronic cases [5]. Infected mammals shed *C. burnetii* in their urine, feces, milk, and birth products [6-8]. Infection can spread both vertically and horizontally, through contact with bodily fluids or transmission through arthropod vectors [6,9].

In dromedary camels, the seroprevalence of *C. burnetii* is reported to range from 0% to 80% [10]. Two studies from Kenya showed different percentages of infected camels; 46% and 18.6% [11,12]. Research work from other countries showed comparable results; 28% in Iran [13,14], 51.6% in Saudi Arabia [15], and 19% in Spain [16]. A recently conducted study in Saudi Arabia highlighted the emergence of *C. burnetii* as a possible cause of uterine infection in dromedary camels [17]. In Egypt, studies concerned with seroprevalence of *C. burnettii* in camels are few. It was diagnosed in 13% of examined animals by immunofluorescence assay (IFA) [18], while using enzyme-linked immunosorbent assay (ELISA), infection was confirmed in 71%, 70% and 40.7% of examined animals; respectively [19,20,21]. Likewise, through molecular tools, *C. burnettii* DNA was diagnosed in 46% of blood samples of examined animals by polymerase chain reaction (PCR) [22].

The isolation of *C. burnetii* is the gold standard for diagnosis of Q fever; however, it is time-consuming and hazardous [23,24]. Due to the absence of characteristic signs for Q fever besides the subclinical and asymptomatic nature in most cases, the seroprevalence studies could be used to indicate exposure and chronicity of infection rather than to detect organism [25]. Detection of antibodies against *C. burnetii* is usually done by ELISA, IFA, or complement fixation test. Due to its higher sensitivity among other practical reasons, ELISA is mostly preferred [26,27].

Molecular-based methods are numerous, and they include nested PCR assay [18,28], real-time PCR [29], touch-down PCR [30], and trans-PCR targeting IS1111, the repetitive transposon-like region of *C. burnetii* [31]. These methods have recently emerged as valuable diagnostic tools, and they can be utilized to study the incidence and prevalence of Q fever and help in understanding its epidemiology.

In Egypt, studies concerned with seroprevalence of *C. burnetii* in dromedary camels are few, and we have no much information regarding its epidemiological status. Therefore, this study was designed to screen for *C. burnetii* infection in camels using quantitative PCR (qPCR) and conventional PCR and to estimate its seropositivity through the detection of anti-*C. burnetii* antibodies using ELISA technique.

## Materials and Methods

### Ethical approval

This study obtained approval from the Ethics Committee of the National Research Centre. Throughout the study, all procedures were carried out in compliance with the Guide for the Care and Use of Laboratory Animals published by the US National Institutes of Health.

### Study design and animals

We conducted a cross-sectional study and included a total of 112 male camels using a convenience sampling strategy. Blood samples were collected from 60 camels at Police Academy and 52 at slaughterhouses in Giza and Cairo Provinces, Egypt. Each camel was subjected to data recording (including disease history, clinical signs, age, breed, and tick infestation) besides molecular and serological screening for *C. burnetii* infection.

### Sampling

We collected blood either from jugular veins of animals at Police Academy or from the cut jugular veins or carotid arteries immediately after slaughter at the slaughterhouses. From each animal, two blood samples (5 ml each) were collected. For molecular studies, ethylenediaminetetraacetic acid-containing Vacutainer tubes were used. For seroepidemiology examination, we used plain Vacutainer tubes to collect samples that were left at room temperature for 12 h to allow clotting and sera separation. The collected anticoagulated whole blood and serum samples were kept at −20°C till used.

### Molecular studies

#### DNA extraction

We extracted that DNA from the collected whole blood samples was using GF-1 Tissue Blood Combi DNA Extraction Kit (SNF, Vivantis, Malaysia) according to the manufacturer’s instructions. The extracted DNA was stored at −20°C till used.

#### Detection of C. burnetii in different samples using SYBR Green real-time PCR

We screened all samples for *C. burnetii* DNA by qPCR using specific primer derived from a transposon-like repetitive region of the *C. burnetii* genome; Trans1 and Trans2 (Table-1) [25]. Briefly, real-time PCR was performed in a final volume of 20 μl, using the 2× QuantiNova^®^ SYBR^®^ Green PCR Master Mix (Cat. no. 208052, Qiagen), 50 pmol of each primer, and 5 µl of extracted DNA. Amplification was carried out in a Stratagene Mx3000P (Agilent Technologies). The cycling profiles were holding step at 95°C for 3 min, 35 cycles of 95°C for 30 s, 62°C for 30 s, and 60°C for 30 s. A single fluorescence reading for each sample was taken at the extension step. Results were expressed by the determination of the cycle threshold, which marked the cycle when the fluorescence of a given sample significantly exceeded the baseline signal. The melting step was set as default in the machine.

**Table-1:** The primers used for PCR and sequencing.

Primer name	Primer sequences	References
Trans gene		
Trans1	5’- TATGTATCCACCGTAGCCAGT C-3’	[25]
Trans2	5’- CCCAACAACACCTCCTTATTC-3’	
CB gene		
CB1	5’- ACTCAACGCACTGGAACCGC-3’	[32]
CB2	5’- TAGCTGAAGCCAATTCGCC-3’	

#### Detection of C. burnetii in different samples using conventional PCR

We subjected all qPCR-positive samples to standard PCR and sequencing. CB1 and CB2 primers were used targeting 257 bp of the superoxide dismutase enzyme coding gene of *C. burnetii* (Table-1) [32]. All PCR amplifications were performed on BIO-RAD Thermal Cycler (BIO-RAD, Singapore) using 2× PCR Master Mix solution (i-Taq, Intron) according to the manufacturer’s recommendation. The protocol of PCR reactions was performed as the following: initial denaturation at 94°C for 5 min and 35 cycles of denaturation at 94°C for 30 s, annealing at 52°C for 1 min and extension at 72°C for 1 min, and then the final extension at 72°C for 5 min [32]. For each reaction, control positive (*C. burnetii* culture) and control negative (without DNA) were used. PCR products were analyzed on 1.5% agarose gel electrophorese with ethidium bromide, and they were visualized by Lab Image software (BIO-RAD, Singapore).

#### Sequencing of PCR products

For PCR product purification, GeneJET Gel Extraction kit (Thermo Fisher Scientific, USA) was used according to the manufacturer’s prescription. The sequencing was conducted using an ABI PRISM^®^ BigDye™ terminator cycle sequencing kits with AmpliTaq^®^ DNA polymerase (FS enzyme; Applied Biosystems), following the protocols supplied by the manufacturer. The obtained sequences were assembled and edited by ChromasPro software (ChromasPro 1.7, Technelysium Pty Ltd., Tewantin, Australia) and the corrected sequences were compared with those available in GenBank by BLAST (https://blast.ncbi.nlm.nih.gov/Blast.cgi).

### Serological studies (detection of *C. burnetii* IgG by ELISA)

Due to the unavailability of species-specific diagnostic reagents, we used sheep *C. burnetii* ELISA kit (GSCIENCE, USA) for the detection of anti-*C. burnetii* IgG antibodies in accordance with the previous studies [11]. ELISA was performed on serum samples following the manufacturer’s recommendations.

## Results

**Figure-1:**
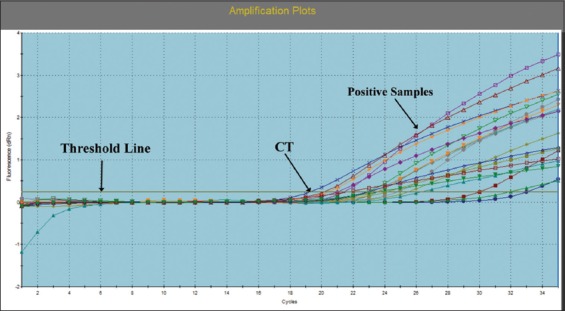
Amplification plots of suspected *Coxiella burnetii* using SYBR Green-based quantitative polymerase chain reaction.

Out of 112 male camels examined, 19 were positive for *C. burnetii* by qPCR (Figure-1) with an overall prevalence of 16.9% (18.6% and 15.1% in Giza and Cairo Provinces, respectively; Table-2). All positive cases were apparently healthy with age range of 10-23 years old.

**Table-2:** The prevalence of Q fever in the studied camels.

Provinces	Number of examined animals	qPCR-positive camels		ELISA-positive camels	
	
		No.	Prevalence (%)	No.	Prevalence (%)
Cairo	53	8	15.1	0	0
Giza	59	11	18.6	5	8.4
Total	112	19	16.9	5	4.5

ELISA=Enzyme-linked immunosorbent assay, qPCR=Quantitative polymerase chain reaction

**Figure-2:**
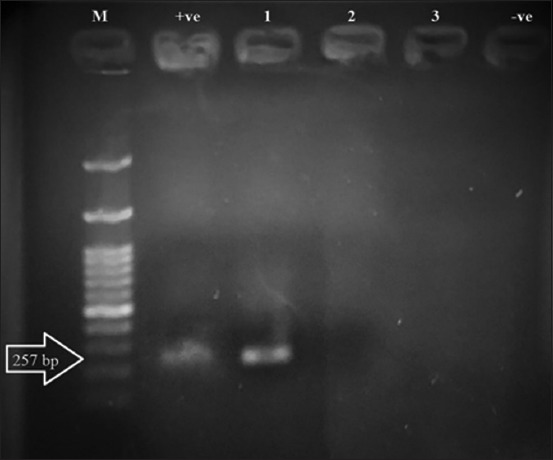
A 1.5% agarose gel electrophoresis of *Coxiella burnetii* polymerase chain reaction using CB1 and CB2 gene. Lane M: 100 bp DNA ladder, lane +ve: control positive, lane −ve: control negative, lane 1 presents 250 bp amplicon of *C. burnetii* positive sample, while lanes 2 and 3 present *C. burnetii* negative samples.

Standard PCR followed by sequencing based on CB1 and CB2 gene identified only two samples as *C. burnetii*. The obtained band of positive samples was around 250 bp in length (Figure-2). Unfortunately, trails for sequencing positive PCR samples revealed poor sequencing.

Only 5 animals (4.5%) were seropositive for anti-*C. burnetii* IgG antibodies. The seropositive cases were among the group selected at Giza Province slaughterhouse (Table-2).

## Discussion

Q fever/coxiellosis is an emerging worldwide arthropozoonosis. Increased awareness of Q fever has been developed recently due to raised frequency of reported outbreaks and economic impact of the disease resulting from loss of animal productivity and herd death [33,34]. Therefore, the estimation of *C. burnetii* prevalence is important to understand the epidemiological status of this disease.

In Egypt, Q fever was reported serologically for the 1^st^ time in 1995 within a risk group of cattle keepers [35]. Later, several studies confirmed the prevalence of the disease in sheep, goat, and cattle livestock [18,20,21,36-38]. However, reports concerning the prevalence and incidence of *C. burnetii* in wild animals, particularly camels, are scarce.

The current study identified *C. burnetii* in dromedary camels using PCR and estimated its seroprevalence using ELISA. Our results revealed that *C. burnetii* was directly identified from whole blood by qPCR in 19 male camels with 16.9% infection rate. This result was in accordance with Mazyad and Hafez [18] who observed *C. burnetii* in 13% of examined camels in Egypt. Comparable results were reported by other studies from Kenya [12], Saudi Arabia [15], Spain [16], and Iran [39] who estimated the prevalence of *C. burnetii* was 18.6, 15.8, 19, and 10.8%, respectively. However, several earlier reports from Egypt [19-22], Chad [40], and Kenya [11] displayed much higher prevalence rates; ranging from 40.7% to 73%. This variance with our result may be due to either difference in geographical and environmental conditions between Egypt and other countries or variations in the sensitivity of laboratory tools. The infected camels in the present study were native breed and appeared clinically healthy at the time of examination, which might be attributed to tolerance developed by the Egyptian camel breed against this disease. Accordingly, camel plays a critical role as reservoir of *C. burnetii* in Egypt.

While qPCR detected *C. burnetii* DNA in camel blood in 16.9% of cases, standard PCR succeeded in amplifying only two samples and failed in sequencing the obtained PCR products. Such results may be clarified by that *C. burnetii* prevalence in blood and milk is lower than faces and urine [15,41]. On the other hand, conventional PCR showed many disadvantages. It is time-consuming, it did not offer quantitative data, and conventional primers showed lack of specificity [42]. Hence, we concluded that qPCR targeting trans-region of *C. burnetii* genome was faster, more sensitive, and valuable than the other primers. This agrees with Costa *et al*. [43] who elucidated that quantitative reverse transcription-PCR (qRT-PCR) can correlate the quantity of DNA with clinical symptoms, and it can be used to follow up treatment and monitor its efficacy. Edvinsson *et al*. [44] demonstrated that qRT-PCR can detect low concentrations of DNA. In our study, the specificity of qRT-PCR was 100%, and it is considered an excellent test.

In this study, we detected IgG antibodies against *C. burnetii* in 5 camels (4.5%) sampled at Giza Province slaughterhouse. This is comparable to an earlier study in the United Arab Emirates that detected a nearby infection rate (7.9%) in camels [45]. The presence of IgG antibodies in ELISA-positive camels indicated exposure to *C. burnetii* in the past and the possibility of chronically harboring the infection by the animals. Consequently, camels may be playing a role in the maintenance of infection in nature.

## Conclusion

Trans-qPCR assay is a rapid, specific, sensitive, automated, and quantitative way of detecting *C. burnetii* infection, especially in early stage. Camels may be playing a critical role in transmission of Q fever to humans in Egypt. Disease awareness between veterinarians, physicians, and camel owners should be raised. Understanding the epidemiology of Q fever and its impact on humans’ health and on the Egyptian economy is of paramount importance. Further research should elucidate further the camels’ role in the transmission of *C. burnetii* to humans and the potential risk factors for exposure.

## Authors’ Contributions

HHAMA, HAH, KAA, AMAB, and YAS participated in the design of the study. HHAMA collected blood samples from camels and sera separation. HHAMA, HAH, and YAS participated in conducting st. PCR and qPCR. HAH, HHAMA, AMAB, and KAA participated in ELISA techniques. HHAMA wrote the first draft of the manuscript. All authors reviewed and approved the final manuscript.
